# Building a Mammalian Retina: An Eye on Chromatin Structure

**DOI:** 10.3389/fgene.2021.775205

**Published:** 2021-10-25

**Authors:** Marwa Daghsni, Issam Aldiri

**Affiliations:** ^1^ Department of Ophthalmology, School of Medicine, University of Pittsburgh, Pittsburgh, PA, United States; ^2^ Department of Developmental Biology, School of Medicine, University of Pittsburgh, Pittsburgh, PA, United States; ^3^ Louis J. Fox Center for Vision Restoration, University of Pittsburgh, Pittsburgh, PA, United States

**Keywords:** progenitors, retina, epigenetics, enhancers, histones, genome organization, neurogenesis, cell fate

## Abstract

Regulation of gene expression by chromatin structure has been under intensive investigation, establishing nuclear organization and genome architecture as a potent and effective means of regulating developmental processes. The substantial growth in our knowledge of the molecular mechanisms underlying retinogenesis has been powered by several genome-wide based tools that mapped chromatin organization at multiple cellular and biochemical levels. Studies profiling the retinal epigenome and transcriptome have allowed the systematic annotation of putative cis-regulatory elements associated with transcriptional programs that drive retinal neural differentiation, laying the groundwork to understand spatiotemporal retinal gene regulation at a mechanistic level. In this review, we outline recent advances in our understanding of the chromatin architecture in the mammalian retina during development and disease. We focus on the emerging roles of non-coding regulatory elements in controlling retinal cell-type specific transcriptional programs, and discuss potential implications in untangling the etiology of eye-related disorders.

## Retinal Development

**FIGURE 1:**
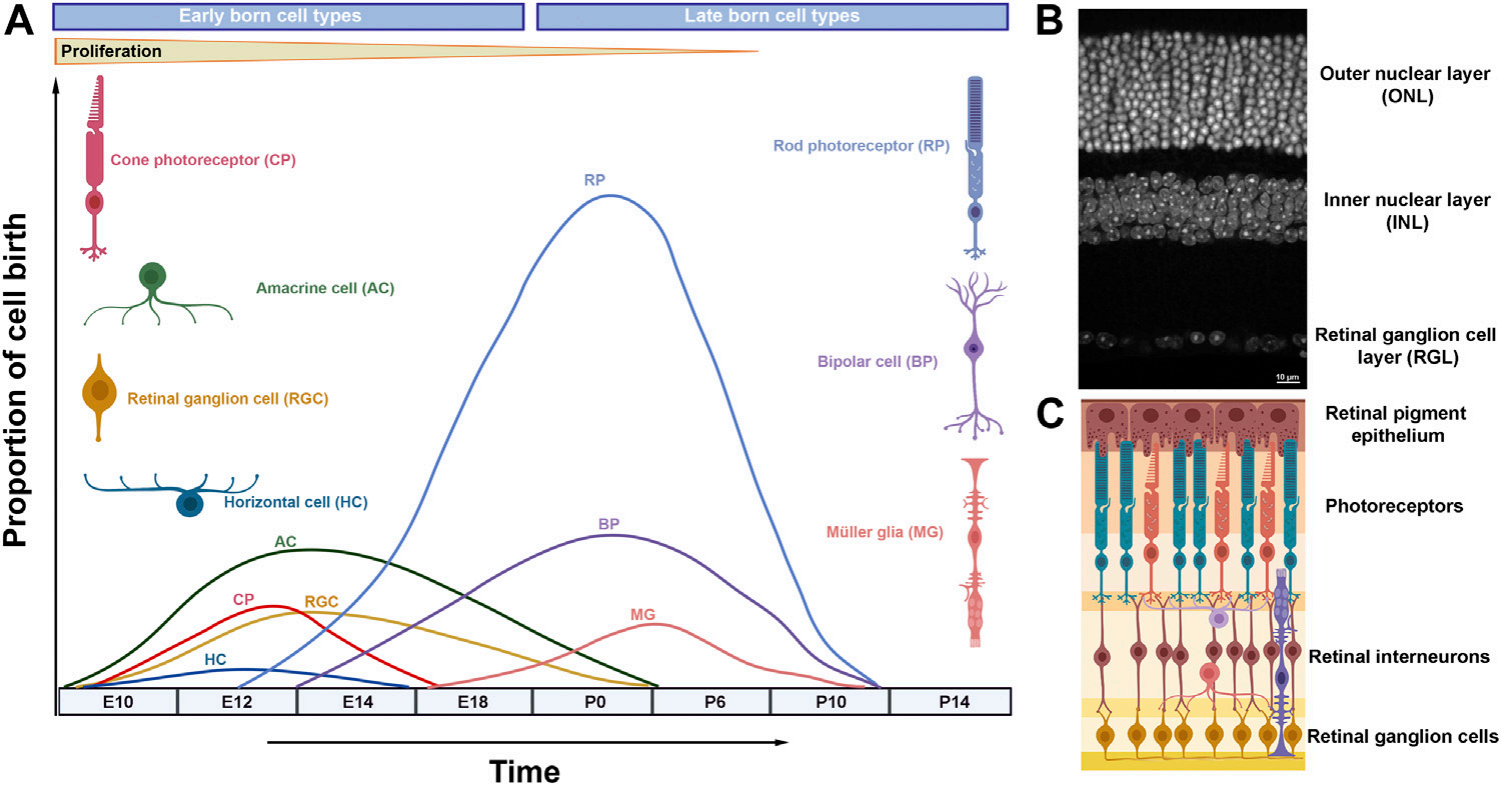
Retinal neurogenesis and organization of the mammalian retina. **(A)** Schematic diagram illustrating waves of retinal neurogenesis and approximate timing of retinal cell type birth. Note that rod photoreceptors, bipolar cells and Müller glia are mainly formed postnatally. **(B)** DAPI staining of the adult mouse retina showing its exquisite laminar structure. **(C)** Retinal laminar position of different cell types.

The retina has been an excellent system to study neurogenesis, due to its simplified anatomical structure, accessibility and well-defined cell types (Agathocleous and Harris, 2009; Demb and Singer, 2015). The vertebrate mature retina contains seven morphologically and functionally distinct cell types, including six types of neurons (ganglion cells, amacrines, bipolars, horizontal cells, and rod and cone photoreceptors) and one type of glia, the Müller glia (Figure 1) (Cepko et al., 1996). Retinal cells are organized into three layers (outer nuclear layer, inner nuclear layer and ganglion cell layer) interconnected by two synaptic layers that facilitate processing of visual signals (Figure 1) (Fisher, 1979). The visual pathway initiates by the response of the photoreceptors to a light stimulus, transducing it into action potentials that propagate to the retinal interneurons (horizontal, bipolar and amacrine cells) and ganglion cells. Eventually the visual input is relayed to the brain through retinal ganglion cell axons that collectively form the optic nerve.

Retinal differentiation initiates when multipotent retinal progenitor cells (RPCs) exit the cell cycle and differentiate into neurons or glia in a temporally conserved order under the control of gene regulatory networks and signaling pathways (Figure 1) (Wetts and Fraser, 1988; Turner et al., 1990; Agathocleous and Harris, 2009). Early retinal development is coordinated by a group of transcription factors (Rax, Otx2, Pax6, Six3, Lhx2, Vsx2 and other) that specifies the eye field within the developing forebrain, promotes retinal proliferation and primes RPCs for subsequent neural differentiation (Zuber et al., 2003). Mutations in many of these genes underlie severe retinal developmental disorders, as observed in microphthalmia (small eye), anophthalmia (absence of the eye), and coloboma (failure in optic fissure closure) cases (Slavotinek, 2011; Reis and Semina, 2015).

Unlike development in the mammalian cortex, retinal cell types are born in waves during which the periods of neuron generation overlap considerably (Figure 1) (Marquardt and Gruss, 2002). Hence, retinal cell types are often classified into early born cell types (ganglion cells, cones, amacrine and horizontal cells) and late born cell types (rods, bipolar cells and Müller glia) (Ohsawa and Kageyama, 2008). Experimental evidence suggests that the ability of retinal progenitors to produce different cell types (competence) changes as development progresses: early progenitors generate early born cell types while late progenitors produce late born cell types (Livesey and Cepko, 2001; Hafler et al., 2012).

The mechanisms that determine RPC competence are rooted in the ability of progenitor cells to integrate signaling pathways and the activities of complex networks of transcription factors (TFs) that drive cell fate decisions at the genomic level (Livesey and Cepko, 2001; Agathocleous and Harris, 2009). Chromatin regulation allows interpretation of identical genomes in a variety of ways, leading to cell type specific transcriptional outputs (Soshnev et al., 2016). Hence, chromatin architecture of the developing retina has been intensively studied, resulting in a wealth of information on transcriptional programs influenced by chromatin regulation during retinogenesis.

Chromatin regulators and retinal lineage-specific programs.

Nuclear DNA is wrapped around a disc of highly conserved proteins (histones) to form the nucleosome, the basic unit of chromatin. Histones are classified into core histones (H2A, H2B, H3 and H4), the principal components of the nucleosome, and linker histones (H1), which bind the nucleosome at the cross point of DNA entry/exit sites (Luger et al., 1997; Vignali and Workman, 1998). Accessibility to DNA requires nucleosome mobilization, which is mediated by large complexes that utilize ATP hydrolysis in the process (Wilson and Roberts, 2011; Kadoch and Crabtree, 2015; Centore et al., 2020). The structural changes in chromatin are often associated with deposition and/or removal of chemical modifications on histone tails, facilitated by distinct multimeric complexes with enzymatic activity (Soshnev et al., 2016; Villasenor and Baubec, 2021).

Given the association between chromatin pathways and regulation of gene expression, genetic studies have focused on investigating the roles of chromatin remodelers and histone modifying complexes during retinal development, a topic that has been reviewed recently (Corso-Diaz et al., 2018; Raeisossadati et al., 2021). Briefly, these studies revealed that chromatin regulators influence retinal progenitor proliferation and cell fate determination in a context-dependent manner. For instance, multiple studies investigated the effect of loss of the polycomb repressive complex 2 (PRC2), which catalyzes the addition of the repressive mark H3K27me3, on retinal development (Aldiri and Vetter, 2009; Aldiri et al., 2013; Iida et al., 2015; Zhang et al., 2015; Yan et al., 2016; Cheng et al., 2018; Fujimura et al., 2018). Mutations in the PRC2 core subunits Ezh2 or Eed lead to reduced retinal proliferation and alteration in neuronal cell fate, particularly amacrine cells, and glia formation (Iida et al., 2015; Zhang et al., 2015; Fujimura et al., 2018). In the postnatal retina, loss of PRC2 function caused photoreceptor degeneration, mediated by a de-repression of the PRC2 targets Six1 and Eya2 (Yan et al., 2016). Meanwhile, perturbation of H3K27me3 removal by knocking down the H3K27me3 demethylase Jmjd3 impacts retinal bipolar cell formation (Iida et al., 2014). Cell-type specific alterations were also observed when MLL, the core subunit of a complex required for mono- and di-methylation of H3K4, was mutated during retinal development. Here, a conditional knockout of MLL impacts retinal proliferation and leads to a progressive loss of horizontal cells in the differentiating retina (Brightman et al., 2018). These examples highlight how chromatin modifying enzymes control multiple aspects of retinal development.

The function of chromatin remodelers that govern nucleosome mobilization has been investigated as well (Das et al., 2007; Lamba et al., 2008; Aldiri et al., 2015). For instance, evidence indicates that Brg1, a core subunit of the SWI/SNF complex, is required for retinal proliferation and photoreceptor differentiation (Aldiri et al., 2015). The effect of Brg1 is likely mediated by its ability to influence the chromatin landscape near actively transcribed cell-type specific genes, as Brg1 predominantly occupies active cis-regulatory elements in the retina, and previous work demonstrated that Brg1 binds transcription factors that drive neurogenesis such as Pax6 and NeuroD1 (Seo et al., 2005; Ninkovic et al., 2013). Additionally, work on cell lines suggests that activities of the enhancer landscape of lineage specification genes is sensitive to the loss of SWI/SNF chromatin remodeling complexes (Aldiri et al., 2015; Alver et al., 2017).

Chromatin-associated complexes can change subunit composition during development, indicative of cell-type-specific roles (Lessard et al., 2007). Indeed, several auxiliary subunits of chromatin regulator complexes are expressed in a stage-specific manner during retinal development but the exact molecular and cellular phenotypes resulting from mutating these proteins during retinogenesis remains to be explored (Lamba et al., 2008; Aldiri et al., 2015).

More recently, chromosome confirmation capture (3C) techniques revealed that manipulation of chromatin regulators such as SWI/SNF and the polycomb repressive complexes can lead to changes in compartment-level chromatin organization (Schoenfelder et al., 2015; Barutcu et al., 2016; Cruz-Molina et al., 2017). These intriguing findings link regulation of gene expression with 3D chromatin architecture *via* activities of chromatin regulators, a function yet to be explored in the retina.

## Epigenetic Landscape Dynamics During Retinogenesis

Genome-wide profiling of histone marks and chromatin associated proteins greatly facilitated the in depth probing of chromatin signature dynamics during developmental stages of mouse and human retina, revealing non-random genomic localization of histone marks and association with gene expression (Popova et al., 2012; Mo et al., 2016; Ueno et al., 2016; Aldiri et al., 2017). In progenitor cells, differentiation genes are poised (H3K27me3-occupied) toward activation and as retinal development proceeds, H3K27me3 is lost and cell type specific genes are expressed (Ueno et al., 2016; Aldiri et al., 2017). Interestingly, the accumulation of H3K27me3 on progenitor genes in differentiated neurons is not as common (Aldiri et al., 2017).

The retinal enhancer landscape exhibits exquisite reconfiguration concomitant with changes in gene expression during retinal developmental transitions: whereas cis-regulatory elements of progenitor genes lose their activities, enhancers targeting differentiation genes are gradually activated (Aldiri et al., 2017). Mechanisms of enhancer potentiation have been the focus of many studies. Current models suggest that priming enhancers for activation during embryonic development can be achieved by a cooperative binding of lineage-specific TFs or by the deployment of a unique set of TFs, termed pioneer factors, that have the ability to bind closed chromatin and facilitate the recruitment of chromatin regulators and lineage-specific TFs and co-factors (Zaret and Carroll, 2011; Drouin, 2014). Retinal pioneer TFs remain poorly characterized but recent genomic data begins to shed light on their roles. For instance, a study examining the genomic profiling of the RPC gene LHX2 reveals global and local reduction of LHX2-bound chromatin accessible sites upon loss of Lhx2, including regulatory regions nearby TFs with potential pioneer function, suggesting that LHX2 functions as a pioneer factor in the developing retina (Zibetti et al., 2019). In another work, analysis of the regulatory elements bound by the photoreceptor differentiation transcription factor Crx in wild type and Crx-mutant retina in mice indicates a limited ability of CRX to remodel chromatin and points toward a cooperative TF binding module in promoting photoreceptor cell fate (Ruzycki et al., 2018). Thus, priming the retinal enhancer landscape during developmental transitions and cell fate choices likely involves multiple mechanisms and is highly context specific.

Mechanisms of enhancer-mediated transcriptional control of genes with multiphasic expression during retinogenesis are particularly interesting, and underscore the complexity of gene regulation. For instance, the transcription factor Sox2 is expressed in RPCs and is confined to amacrine cells and Müller glia in adult retina (Taranova et al., 2006). In principle, such a complex temporal and spatial expression pattern can occur *via* recruitment of stage- and cell-type specific TFs and/or by the utilization of cell-type exclusive enhancers. Retina-specific enhancer elements with temporally restricted activities have been identified as the case with those nearby Otx2, a transcription factor expressed in a subset of progenitor cells and marks bipolar cells and photoreceptors (Emerson and Cepko, 2011; Kaufman et al., 2021). Notably, Sox2 chromatin architecture has been studied given its essential roles in maintaining stem cell pluripotency, revealing a complex regulatory landscape with multiple putative enhancer elements, including stem cell-specific regulatory constituents that are essential for Sox2 expression (Li et al., 2014; Zhou et al., 2014; Bonev et al., 2017).

Interestingly, downregulation of Sox2 in rod photoreceptors is accompanied by site-specific deposition of the repressive histone mark H3K27me3 (Norrie et al., 2019). Whereas Sox2 coding region and nearby enhancers are occupied by H3K27me3, Sox2-regulatory elements that are hundreds of base pairs away holds limited levels. This implies that not all regulatory elements are created equally and underscores a locus-specific utilization of repressive mechanisms on enhancer elements. Florescence *in situ* hybridization (FISH) performed on rod nuclei indicates that while Sox2 coding region is located in euchromatin, its long-range putative enhancers reside in heterochromatin, thus likely inaccessible to the action of repressive complexes (Norrie et al., 2019). These data are in agreement with the finding that rod photoreceptors render a substantial fraction of vestigial regulatory elements (enhancers that used to be active in earlier stages of retinogenesis) inaccessible to repression mediated by DNA methyltransferase (Mo et al., 2016).

Diverse histone marks tend to co-exist, leading to an excessive number of possible combinatorial readouts and renders interpretation of epigenomic maps challenging. To facilitate a better understanding to the biological roles of combinations of histone marks and chromatin associated proteins, a computational modeling that utilizes machine learning algorithms (ChromHMM) was developed to distinguish groups (states) of co-occurring chromatin marks across the genome (Ernst and Kellis, 2010; 2012). Applying this method to ChIP-Seq data generated from mouse and human developing retina led to the identification of several chromatin states that capture known genomic elements such as active promoters and enhancers, insulators and repressed regions (Aldiri et al., 2017). ChromHMM analysis was also informative in exploring prevailing chromatin states in retinoblastoma and retinal organoids (Hiler et al., 2015; Aldiri et al., 2017). Later, a computational work that integrates retinal chromatin states and 3D FISH imaging successfully predicted genome-wide euchromatin and heterochromatin compartmentalization in the mouse retina (Norrie et al., 2019).

Mapping of the epigenomic marks and regions of chromatin accessibility has emerged as a powerful tool to annotate retinal putative regulatory elements, particularly enhancers (Wilken et al., 2015; Aldiri et al., 2017; Hughes et al., 2017; Wang et al., 2018; Cherry et al., 2020; Xie et al., 2020). Given the essential roles of enhancer elements in controlling cell type specific differentiation programs during retinogenesis, we will discuss recent progress in the field and highlight examples related to the gene regulatory networks controlling retinal cell fate choices.

## Discovery of Retinal Enhancers

Enhancers are stretches of non-coding DNA elements that spatially and temporally regulate transcription by acting as platforms to recruit transcription factors and transcriptional machinery, irrespective of sequence orientation (Gasperini et al., 2020). Enhancers are the main source for communication between chromatin and the environment as they contain motifs that can bind transcription factors and recruit effectors of signaling pathways (Long et al., 2016). Biochemically, enhancers are characterized by occupancy of active histone marks (i.e., H3K27ac and/or H3K4me1) and chromatin-associated proteins (i.e., p300), overlaying areas of open chromatin (Visel et al., 2009; Creyghton et al., 2010; Rada-Iglesias et al., 2011; Thurman et al., 2012). Interestingly, while H3K27Ac has been widely validated as a hallmark for active enhancers in the animal kingdom, association of H3K27Ac deposition with active regulatory elements in plants appears species-specific (Yan et al., 2019).

Recent advances in techniques that map 3D genome organization demonstrated that enhancers may act over long genomic distances, *via* looping, to contact their cognate gene promoters in 3D space (Li et al., 2012; Fang et al., 2016; Mumbach et al., 2016). The prevailing model is that a promoter-enhancer interaction mediates activation of gene expression by bringing transcription factors and transcription machinery into promoter proximity (Robson et al., 2019). However, whether promoter-enhancer physical contact is a universal prerequisite mechanism for gene activation is not firmly established (Chen et al., 2018; Benabdallah et al., 2019; Crump et al., 2021). There are hundreds of thousands of putative regulatory elements in the human genome, far in excess of number of genes, underscoring the complexity of enhancer function in organ development and homeostasis.

Classically, strategies to pinpoint cell-type specific cis-regulatory elements in the developing retina have exploited DNA conservation and enrichment of lineage-specific transcription factor motifs coupled with *in vivo* screening for enhancer activities. This method was successful in the identification of numerous distal regulatory elements near genes essential for retinal development and cell-type specification such as Vsx2 and Grm6 (bipolar cells), Nrl, Otx2 and Prdm1 (photoreceptors), Atoh7 (ganglion cells), Onecut1 and Thrb (cones/horizontal cells), and Pax6 (RPCs, amacrine cells), among others (Kleinjan et al., 2004; Rowan and Cepko, 2005; Riesenberg et al., 2009; Willardsen et al., 2009; Emerson and Cepko, 2011; Kautzmann et al., 2011; Emerson et al., 2013; Mills et al., 2017; Goodson et al., 2020; Patoori et al., 2020).

Comparative genomics employing convergent evolution were also useful in identifying and characterizing putative retinal enhancer elements (Kvon et al., 2016; Partha et al., 2017; Roscito et al., 2018). The logic behind this interesting method is that regulatory elements that are essential for vision are under evolutionarily constraints to preserve visual structures and functions. In animals where vision is regressed, such as subterranean mammals, vision-related regulatory regions and/or their target genes undergo accelerated mutation rate and suffer sequence divergence due to relaxed evolutionally constraints, thus revealing DNA sequences potentially essential for development of optical structures. Such a strategy was employed to investigate enhancer elements in the ground-dwelling moles, leading to the identification of several retina-specific regulatory regions associated with vision deterioration, including those nearby Pax6 (Partha et al., 2017). Still, not all regulatory elements are conserved at the DNA level, and many highly conserved enhancers lack *in vivo* activities in transgenic assays (Pennacchio et al., 2013). Thus, complementary approaches to profile the cis-regulome remain essential to elucidate enhancer structure and function.

With the broad availability of next generation sequencing platforms, profiling chromatin structure in the developing retina has taken a momentum, facilitating the discovery of genome wide putative distal enhancers with a relative ease. Taking advantage of transcription factors occupancy as a proxy to the identification of distal enhancer regions, numerous transcription factors involved in retinal cell fate choices have been surveyed using ChIP-Seq and, more recently, CUT and RUN, including OTX2, ATOH7, NRL, CRX, MEF2D, RORB and LHX2 (Corbo et al., 2010; Swaroop et al., 2010; Samuel et al., 2014; Andzelm et al., 2015; Zibetti et al., 2019; Cherry et al., 2020; Brodie-Kommit et al., 2021). Likewise, histone modifications associated with active promoters and enhancers have been extensively charted in the developing retina, and hundreds of cis-regulatory elements have been catalogued (Popova et al., 2012; Wilken et al., 2015; Aldiri et al., 2017; Hughes et al., 2017; Xie et al., 2020). More recently, a transcriptional profiling of non-coding RNAs, often transcribed from active enhancer regions, was performed to delineate cone and rod regulatory elements in wild type and Nrl mutant mice (Perez-Cervantes et al., 2020).

Chromatin accessibility has become a popular method to identify cis-regulatory elements. Studies on bulk tissues from human and murine developing retina revealed temporal dynamics in chromatin accessibility associated with changes in gene expression during retinogenesis. Earlier work utilized DNaseI hypersensitivity (DHS) to profile the mouse developing retina, leading to the identification of developmentally regulated enhancer elements near the transcription factors Neurog2, Otx2 and Olig2, (Wilken et al., 2015). By far, assay for transposase-accessible chromatin with sequencing (ATAC-Seq) has become the most common technique used to profile regulatory elements, revealing enhancer landscape dynamics in the mouse and human developing retina (Mo et al., 2016; Aldiri et al., 2017; Hughes et al., 2017; Cherry et al., 2020; Xie et al., 2020). As retinal organoids become a powerful method to investigate and model retinal development and disease (Eiraku et al., 2011; Volkner et al., 2016), ATAC-Seq was used to demonstrate a high temporal correlation of regulatory landscape dynamics in retinal organoids and human fetal retina, further validating retinal organoids as a robust model to study human retina (Xie et al., 2020).

To date, most of the studies surveying retinal open chromatin regions used bulk tissues as input, which renders the determination of cell type specific deployment of regulatory elements in rare retinal cell types challenging. To overcome this limitation, ATAC-Seq, and sometimes ChIP-Seq, experiments have been performed on purified cells from transgenic mice carrying cell type-specific reporter genes, and as a result, epigenomic data from enriched rods, cones, bipolar cells and Müller glia are now available (Mo et al., 2016; Ueno et al., 2016; Hughes et al., 2017; Ueno et al., 2017; Murphy et al., 2019; VandenBosch et al., 2020).

The advent of single cell technologies, methods that circumvent heterogeneity and allow the investigation of rare cell populations at high resolution, has revolutionized the field, and a large cohort of studies focusing on surveying the adult and developing retinal single cell transcriptome has been performed (Clark et al., 2019; Kim et al., 2019; Liang et al., 2019; Menon et al., 2019; Cherry et al., 2020; Lu et al., 2020; Sridhar et al., 2020; Brodie-Kommit et al., 2021; Wu et al., 2021). Still, matching studies that investigate retinal chromatin accessibility dynamics at the single cell resolution remain limited (Xie et al., 2020). With recent technical advances that enable the simultaneous profiling of transcriptome and epigenome from the same cells, it is almost certain that work is underway to accurately outlining the epigenome dynamics in relation to gene expression in retinal cell populations (Kashima et al., 2020; Weir et al., 2021).

## Functional Validation of Retinal Enhancers

Genome-wide analysis to delineate putative regulatory elements is a robust method to infer enhancer activities but not without limitations (Halfon, 2019). With the wealth of information available on the genomic location of predicted retinal enhancers, derived primarily by biochemical annotations and computational methods, *in vivo* experimental characterization of those elements remains necessary to validate their functions. In theory, a regulatory element should recapitulate its cognate gene’s spatial and temporal expression pattern, and when mutated should lead to alteration in gene expression. A large body of work has been directed toward investigating enhancer activities in the retina using reporter assays, which test the ability of a candidate enhancer sequence to activate a reporter gene (i.e., GFP, LacZ and luciferase). Electroporation of the mouse developing retina has been the main method for construct introduction into the retina, testing enhancer activity one element at a time (Montana et al., 2011; Wang et al., 2014; Goodson et al., 2020; Kaufman et al., 2021). *In vivo* transgenesis using mouse, zebrafish and the frog xenopus was also used (Hutcheson et al., 2005; Ghiasvand et al., 2011; Fang et al., 2017; Bhansali et al., 2020; Kaufman et al., 2021). High throughput strategies to interrogate the activities of retinal enhancers has been explored as well. In one such a study, massively parallel reporter assay (MPRA) was used to investigate photoreceptor cis-regulatory elements bound by CRX (White et al., 2013).

CRISPR-based genome editing technology has tremendously facilitated testing the function of retinal enhancers *in vivo* by providing a venue to efficiently delete non-coding regions with precision (Osterwalder et al., 2018). Emerging studies on enhancer elements nearby Vsx2, Otx2 and Prdm1 in retinal explants and mouse knockouts uncovered lineage- and stage-specific regulatory elements important for photoreceptor and bipolar cell fates (Wang et al., 2014; Norrie et al., 2019; Chan et al., 2020; Goodson et al., 2020; Kaufman et al., 2021). Still, whether an enhancer is required for the expression of its target gene remains challenging to address given the complex nature of the chromatin landscape. Enhancers may regulate the expression of a single target (i.e., a single or many enhancers, one target gene) or acting promiscuously on multiple genes (i.e., one enhancer, multiple target genes). As such, *in vivo* perturbations of regulatory elements, especially those nearby functionally important genes, may result in no molecular or cellular consequences, likely due to enhancer redundancy (Kurokawa et al., 2004; Osterwalder et al., 2018). Additionally, an enhancer may govern the expression of gene(s) broadly expressed in multiple tissues during embryogenesis, leading to pleiotropic effects and/or embryonic lethality upon loss of enhancer function. Still, enhancer deletion assays remain an important tool to reveal molecular mechanism underlying biological functions of enhancer landscape.

### Super enhancers and retinal cell type specific programs

Developmentally critical transcription factors are often marked with strong enhancers to drive and/or maintain robust expression. Work on embryonic stem cells defined a subclass of regulatory elements, termed super-enhancers (SEs), that are selectively enriched near genes important for stem cell identity (Whyte et al., 2013). Super-enhancers tend to span large genomic regions and are strongly enriched in mediator complex and transcription factors, particularly those driving lineage-specific programs (Parker et al., 2013; Adam et al., 2015). The importance of SE size is not clear, but it was proposed that strong H3K27Ac occupancy that demarcates these regulatory clusters weakens DNA-histone interactions, thus exposing DNA to transcription factors (Parker et al., 2013). Evidence suggests that SEs drive high levels of transcriptional activity and are particularly sensitive to perturbations (Hnisz et al., 2013; Loven et al., 2013; Whyte et al., 2013; Bahr et al., 2018). Recent studies propose that transcription factors, activators and co-activators occupying SEs form condensates with liquid-phase separation properties (Hnisz et al., 2017; Boija et al., 2018; Sabari et al., 2018). However, the functional significance of SEs and whether a super-enhancer constitutes a single functional unit of cooperating regulatory clusters or a mere stretch of aggregated enhancers remains unclear (Pott and Lieb, 2015; Moorthy et al., 2017).

Given the emerging interest in SEs roles in regulating tissue-specific gene expression, dynamically regulated SEs in mouse and human developing retina have been annotated (Aldiri et al., 2017). Studies that functionally investigate SEs in the retina remain limited but available data suggest central roles in driving retinal cell fate choices. For example, a large deletion (35 kb) in an area that overlaps a super-enhancer nearby Vsx2 caused a complete loss of retinal bipolar cells, while proliferation appears to proceed normally (Norrie et al., 2019). This regulatory region contains conserved elements that can drive reporter expression in RPCs, Müller glia and bipolar cells (Rowan and Cepko, 2004; Kim et al., 2008), and a recent study demonstrated that knocking down of a smaller portion of the Vsx2 SE also impacts bipolar cell differentiation (Goodson et al., 2020). Thus, while the concept of super-enhancers is appealing, detailed functional studies are needed to elucidate the exact biological roles of SEs and their constituents in promoting retinal cell fate acquisitions.

## Retinal Enhanceropathies

Defining cis-regulatory elements is crucial to understand disease mechanisms, as variations in DNA sequences linked to inherited human disorders often lie in non-coding regions (Chatterjee and Ahituv, 2017). Retinal diseases associated with alterations in regulatory landscape have been reported but only in a handful of cases has a causative link been suggested. A clear example illustrating a direct role of regulatory elements in inherited retinal disorders comes from studies on patients with nonsyndromic congenital retinal nonattachment (NCRNA), an autosomal recessive retinal disease characterized by congenital blindness due to loss of RGCs and optic nerve atrophy (Keser et al., 2017). A deletion in a non-coding DNA region 20 kb upstream of the proneural bHLH transcription factor ATOH7 has been linked to the disease (Ghiasvand et al., 2011). Transgenic reporter assays in mouse and zebrafish demonstrated that this non-coding element has developmental activities that matched the spatiotemporal expression of Atoh7, suggesting that it acts as an enhancer element for Atoh7 (Ghiasvand et al., 2011). Subsequent studies identified pathogenic mutations in the ATOH7 coding region itself, further linking NCRNA to misregulation of Atoh7 (Keser et al., 2017; Kondo et al., 2018). Surprisingly, deleting the orthologous murine enhancer region does not recapitulate the disease phenotype, suggesting a differential biological significance of mouse and human Atoh7 enhancer landscape (Miesfeld et al., 2020). Other examples that identified variations in enhancer elements with links to ocular disorders include those nearby Pax6 (aniridia) and Samd7 (retinitis pigmentosa) (Bhatia et al., 2013; Van Schil et al., 2016).

Global alterations in retinal enhancer landscape have been observed in patients with retinal degenerative diseases. A recent study profiled the genome-wide chromatin accessibility in patients with dry age-related macular degeneration (AMD), a disease characterized by a progressive loss of photoreceptors, and revealed a genome-wide quantitative reduction in chromatin accessibility associated with advanced stages of the disease, particularly in the macular region (Wang et al., 2018). Of note, the genomic regions that recruit gene regulatory networks controlling photoreceptor gene expression seems to be most impacted in those patients (Wang et al., 2018).

Retinal diseases can be associated with genomic rearrangements that lead to the formation of a *de novo* regulatory landscape, causing gene deregulation. In one such instance, a cohort of patients with autosomal-dominant retinitis pigmentosa suffered a structural rearrangement that led to a repositioning of retina-specific regulatory landscape nearby GDPD1, a gene involved in lipid metabolism. The ectopic activation of GDPD1 driven by the newly created enhancer region likely leads to de-regulation of lipid metabolism, an essential process for phototransduction (de Bruijn et al., 2020; Fu et al., 2021). This work demonstrates how recent advances in surveying 3D genome organization can facilitate the discovery of molecular mechanisms underlying retinal diseases.

### Retinal 3D Nuclear Organization and High Order Chromatin

**FIGURE 2:**
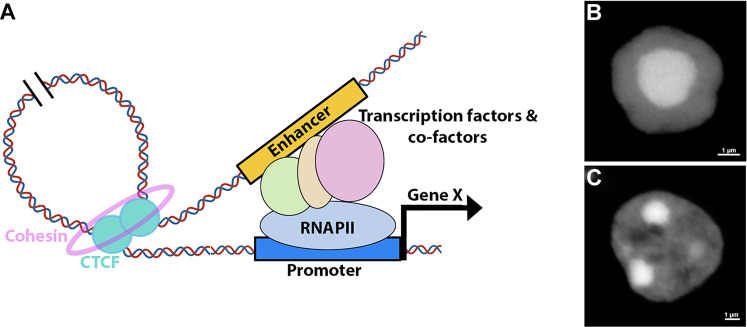
Retinal 3D nuclear organization. **(A)** Model for enhancer-mediated activation of gene expression. The process involves formation of a DNA loop, facilitated by the recruitment of CTCF and cohesin, that brings distal enhancers into proximity of the promoters. Enhancers enable the recruitment of transcription factors, co-factors and transcriptional machinery to the promoter. **(B**,**C)** Nuclear structure of murine rod **(B)** and bipolar **(C)** cells as revealed by DAPI staining.

Thanks to the rapid development of 3C techniques, the mammalian 3D genome conformation has been profiled at high resolution, illuminating that chromatin is organized into compartments in which multiple levels of DNA-DNA preferential interactions exist (Dixon et al., 2012; Lieberman-Aiden et al., 2009). At the chromosome level, transcriptionally active and inactive regions are spatially segregated into very large genomic regions, called compartments A and B, respectively (Lieberman-Aiden et al., 2009). Within each compartment distinct territories, the topologically associated domains (TADs), exist in which promoter-enhancer contacts are heavily constrained (Dixon et al., 2012; Rao et al., 2014). How hierarchical genomic folding is formed and maintained is under intensive investigation, but evidence points toward a major role of the transcription factor CTCF (Merkenschlager and Nora, 2016; Nora et al., 2012). Current models propose that genomic contacts are established *via* loop formation that involves homo-dimerization of CTCF at the loop anchors (Figure 2) (Rao et al., 2014; Hnisz et al., 2016; Weintraub et al., 2017). These interactions are further stabilized by a cohesin complex that forms a ring around the loop anchor region (Kagey et al., 2011; Hnisz et al., 2016; Merkenschlager and Nora, 2016; Rao et al., 2017; Weintraub et al., 2017). The orientation of CTCF binding seems to be important for the proper formation of the loop (de Wit et al., 2015; Guo et al., 2015).

Research investigating high order chromatin of the developing retina remains limited, and available data is primarily collected from bulk mouse retina and purified rod photoreceptors (Falk et al., 2019; Norrie et al., 2019; Tan et al., 2019). Overall, retina hierarchical genomic organization is similar to what has been reported in other tissues, including in the cortex, and developmental transitions of compartments A and B correlated well with retinal chromatin signature (Dixon et al., 2012; Norrie et al., 2019). However, while the number of TADs remain relatively constant as neural progenitors differentiate into cortical neurons, rod photoreceptors have significantly more TADs than in RPCs or cortical neurons, presumably due to the compact nature of rod nuclei (Bonev et al., 2017; Norrie et al., 2019).

Genomic technologies have enabled the identification and cataloging of putative regulatory elements yet defining their cognate genes remains challenging (Buecker and Wysocka, 2012; de Laat and Duboule, 2013). Promoter-enhancer contacts are generally difficult to identify using Hi-C due to resolution limitations but work on developing neural tissues captured the dynamics of several prominent interactions associated with genes important for neurogenesis (Bonev et al., 2017; Norrie et al., 2019). This is illustrated by Sox2 locus, where changes in the Sox2 expression during cortical and retinal differentiation is associated with re-wiring of longs-range contacts between Sox2 promoter and regulatory elements hundreds of kilobases away (Bonev et al., 2017; Norrie et al., 2019).

It is now broadly accepted that enhancers can act over great genomic distances, *via* CTCF-mediated looping, to regulate promoter activities, bypassing proximally located genes (Schoenfelder and Fraser, 2019). The specific roles of CTCF in retinal differentiation remain unclear but early studies on chick retina suggest regulatory functions associated with Pax6 (Li et al., 2006). CTCF is essential for proper retinal formation as loss of CTCF expression in the murine developing retina leads to massive cell death (Watson et al., 2014). The genome wide occupancy of CTCF in the developing retina has been profiled, revealing constitutive and dynamic CTCF occupancy across retinal genome during retinogenesis (Aldiri et al., 2017). Interestingly, work on retinal organoids suggest that maintaining a robust CTCF binding memory in stem cells reprogrammed from rod photoreceptors is important for efficient differentiation of retinal organoids (Hiler et al., 2015). Still, evidence from stem cells indicates that global loss of chromatin loops has a minimal effect on gene expression (Zuin et al., 2014; Rao et al., 2017). Thus the retina-specific roles of CTCF likely reflect gene-specific regulatory functions independent of 3D genome structure, although more work is needed to examine this idea.

### Inverted Nuclear Architecture in Mouse Rod Photoreceptors

The chromatin spatial architecture is commonly shared among animal nuclei, where inactive heterochromatin is preferentially sequestered to the nuclear periphery while active euchromatin occupies the nuclear interior (Holla et al., 2020; Solovei et al., 2016). The structure of rod photoreceptor nuclei in nocturnal animals has deviated from this organization: heterochromatin is densely concentrated in the nuclear center while euchromatin occupies the outer edges (Figure 2) (Solovei et al., 2009). Data suggest that the inverted nuclear arrangement in rods reduces light scattering, effectively converting the nuclei into micro-lenses that enhance vision in dim light conditions (Solovei et al., 2009; Solovei et al., 2013; Subramanian et al., 2019). As such, this inverted nuclear structure in rods represents a clear example of how 3D nuclear architecture may directly influence a physiological function. Still, inverted nuclei structure is also observed in other cell types such as olfactory sensory neurons and neutrophils but the exact biological purpose of this organization in these cells is not clear (Clowney et al., 2012; Solovei et al., 2013).

Despite the stark structural differences between inverted and conventional nuclei, Hi-C data indicate that the hierarchical chromatin compartmentalization is qualitatively similar (Falk et al., 2019; Tan et al., 2019). Additionally, studies integrating Hi-C experiments with computational modeling suggest that the spatial partitioning of heterochromatin and euchromatin in both conventional and inverted nuclei is mediated by liquid-phase separation dynamics, driven primarily by heterochromatin interactions (Falk et al., 2019; Tan et al., 2019).

The establishment of inverted nuclei occurs during rod photoreceptors terminal differentiation and is completed by postnatal day 28 in mice (Solovei et al., 2009). During this process, rod precursor nuclei experience morphological reorganization where chromocenters gradually dissociate from the nuclear periphery and coalesce centrally (Solovei et al., 2009). At the molecular level, nuclear inversion is correlated with loss of LBR and Lamin A/C, proteins essential for tethering heterochromatin to the nuclear periphery (Clowney et al., 2012; Solovei et al., 2013). The molecular mechanism involving downregulation of lamina-associated proteins during rod differentiation has not been fully explored but preliminary evidence suggests a role for the transcription factor Casz1 in association with polycomb proteins in repressing Lamin A (Mattar et al., 2018). Casz1 is also expressed in cone photoreceptors and does not seem to regulate LBR expression (Mattar et al., 2018). Thus, it is likely that repression of lamina-associated proteins in differentiating rods involves other rod-specific transcription factors (Hughes et al., 2017; Mattar et al., 2018). Interestingly, while loss of LBR can alter the nuclear structure, it does not affect global gene expression (Solovei et al., 2013; Norrie et al., 2019).

## Concluding Marks

Genomic studies thus far have provided insights into modulation of retinal development by chromatin structure, yet the field is still in its infancy and a tremendous amount of work is needed to gain a comprehensive understanding on how epigenetics shape retinal development and are associated with retinal diseases. As sequencing technologies and computational analyses continue to rapidly evolve, it is likely that more high resolution data from retinal cell types will be available in the near future.

What are the long-range interactions that occur among cis-regulatory elements during retinal development and how essential are they to retinal development and homeostasis? Are these interactions disrupted in ocular diseases? If so in what way? What are the factors that govern nuclear organization in retinal neurons? How does nuclear architecture influence gene expression during retinal cell type specification? Do liquid-phase separation properties of nuclear compartments influence retinal transcriptional programs? These are some of the outstanding questions that are likely to help elucidating how chromatin influence transcriptional regulation in the retina.

Animal models have been immensely valuable in understanding molecular mechanisms underlying human biology and diseases but more studies investigating chromatin structure in human native and diseased retina are needed. This is particularly important to advance therapeutic strategies aiming at stimulating regeneration and/or preventing degeneration in the mammalian retina.
